# The Role of Mucin Expression in the Diagnosis of Oesophago-Gastric Cancer: A Systematic Literature Review

**DOI:** 10.3390/cancers15215252

**Published:** 2023-11-01

**Authors:** Nikhil Manish Patel, Georgios Geropoulos, Pranav Harshad Patel, Ricky Harminder Bhogal, Kevin Joseph Harrington, Aran Singanayagam, Sacheen Kumar

**Affiliations:** 1 The Royal Marsden NHS Foundation Trust, London SW3 6JJ, UK; 2 The Upper Gastrointestinal Surgical Oncology Research Group, The Institute of Cancer Research, London SW7 3RP, UK; 3 Division of Radiotherapy and Imaging, The Institute of Cancer Research, London SW7 3RP, UK; 4 Centre for Molecular Bacteriology and Infection, Imperial College London, London SW7 2AZ, UK; 5 Department of Upper Gastrointestinal Surgery, Digestive Disease & Surgery Institute, Cleveland Clinic London Hospital, London SW1X 7HY, UK

**Keywords:** mucins, early diagnosis, oesophago-gastric cancer, oesophageal adenocarcinoma, oesophageal squamous cell carcinoma, gastric adenocarcinoma

## Abstract

**Simple Summary:**

Oesophago-gastric cancers are associated with poor survival due to late diagnosis. By the time patients present to their doctor, there is a high chance the disease has spread around the body. Currently, less than 40% of patients with oesophago-gastric cancer can be treated with potentially curative intent. Non-invasive testing of patients who are at higher risk of oesophago-gastric cancer may help to identify these cancers earlier, before they have spread around the body, potentially increasing chances of cure. Mucins are proteins that are found throughout the digestive system and involved in the development of this disease. In this study, the literature on the role of mucins in the diagnosis of oesophago-gastric cancer has been reviewed. Mucins MUC1, MUC2, MUC5AC and MUC6 were the most frequently implicated in oesophago-gastric cancer. Further study of these mucins in high-risk populations may reveal new markers for non-invasive early diagnostic testing for oesophago-gastric cancer.

**Abstract:**

Survival in oesophago-gastric cancer (OGC) is poor due to early diagnostic challenges. Non-invasive risk stratification may identify susceptible patients with pre-malignant or benign disease. Following diagnostic confirmation with endoscopic biopsy, early OGC may be treated sooner. Mucins are transmembrane glycoproteins implicated in OGC with potential use as biomarkers of malignant transformation. This systematic review defines the role of mucins in OGC diagnosis. A literature search of MEDLINE, Web of Science, Embase and Cochrane databases was performed following PRISMA protocols for studies published January 1960–December 2022. Demographic data and data on mucin sampling and analysis methods were extracted. The review included 124 studies (*n* = 11,386 patients). Gastric adenocarcinoma (GAc) was the commonest OG malignancy (*n* = 101) followed by oesophageal adenocarcinoma (OAc, *n* = 24) and squamous cell carcinoma (OSqCc, *n* = 10). Mucins MUC1, MUC2, MUC5AC and MUC6 were the most frequently implicated. High MUC1 expression correlated with poorer prognosis and metastases in OSqCc. MUC2 expression decreases during progression from healthy mucosa to OAc, causing reduced protection from gastric acid. MUC5AC was upregulated, and MUC6 downregulated in GAc. Mucin expression varies in OGC; changes may be epigenetic or mutational. Profiling upper GI mucin expression in OGC, with pre-malignant, benign and healthy controls may identify potential early diagnostic biomarkers.

## 1. Introduction

Oesophageal (OC) and gastric cancers (GC) have been identified as ‘cancers of unmet need’ due to their late diagnosis and subsequent poor survivability [1]. Diagnosing these malignancies early to enhance the chances of curing patients is challenging, and, consequently, the 5-year survival rates of these diseases are reported to be as low as 15.0% for OC and 21.6% for GC in the United Kingdom (UK) [2]. Improving the potential for cure is a challenge on a global scale as GC and OC are the third and sixth highest causes of death, respectively [3].

Due to the expansile nature of the oesophagus and stomach, early cancers rarely present with upper gastrointestinal (GI) symptoms. The typical ‘red flag’ symptoms, which include dysphagia, vomiting, weight loss and anaemia, that trigger patients to consult their doctors, and, subsequently, undergo investigations such as oesophago-gastro-duodenoscopy (OGD) are often indicative of advanced disease. Therefore, after eventually presenting to a medical professional, only 39.2% of patients are suitable for treatment with curative intent after a formal diagnosis of oesophago-gastric cancer (OGC) [4,5,6]. 

Early diagnosis, in conjunction with multi-modal therapy, is crucial for affording patients with these cancers a better chance at survival [5]. Although this may include chemotherapy or recruitment to clinical trials if appropriate, earlier diagnosis could allow a higher proportion of patients to undergo potentially curative treatment, including perioperative chemotherapy and radical surgery [6]. 

### 1.1. Current Technologies in Early Diagnosis of Oesophago-Gastric Cancer

Early diagnosis could be achieved through risk-stratification testing, which could crucially identify patients susceptible to or with early and potentially curable OGC, who would then undergo OGD and biopsy to confirm the diagnosis and obtain tissue to plan definitive treatment. Currently, there are no risk-stratification tests available in mainstream practice, and, consequently, the assessment of clinical factors is a mainstay of estimating the risk of potential OG malignancy. Recognised carcinogens include smoking and alcohol in oesophageal squamous cell carcinoma (OSqCc), gastro-oesophageal reflux disease (GORD) is associated with increased risk of oesophageal adenocarcinoma (OAc), and Helicobacter pylori predisposes patients to gastric cancer (GC) [7,8]. However, a number of different technologies are in developmental phases with the aim of achieving early diagnosis in OGC. One example is the Cytosponge-trefoil factor 3 test; a non-endoscopic cell collection device with an immunohistochemical biomarker is used to diagnose Barrett’s metaplasia, the pre-malignant precursor lesion to oesophageal adenocarcinoma (OAc) [9]. The aim is for this device to be established in the community, to identify patients, who are more susceptible to OAc, so that they are prioritised for endoscopic surveillance. However, this device has been met with variable acceptability among trial populations, due to discomfort experienced when attempting to swallow the Cytosponge and in extracting it from the oesophagus by pulling on the string attached to it [10]. Furthermore, this device may only be able to detect potential Barrett’s metaplasia and adenocarcinoma limited to the oesophagus and cardia of the stomach alone, and not more distal gastric or fundal cancers, limiting its use.

In comparison, patients may be more likely to engage with non-invasive testing methods, which may be less uncomfortable and easier to adhere to, leading to greater compliance and yield of results. This would enable the test to be adopted by larger populations to afford the benefits of early cancer diagnosis on a greater scale; identifying more patients at risk of disease. One such example involves the analysis of volatile organic compounds (VOCs) as diagnostic biomarkers of OGC in exhaled breath and urine samples [11,12,13]. Exhaled breath analysis has been well established for diagnosing Helicobacter pylori infection: a carcinogen for GC, small intestinal bacterial overgrowth and asthma. However, testing for VOCs in exhaled breath and biofluids (e.g., urine) is yet to be proven as a reliable and reproducible method for identifying those at risk of OGC.

### 1.2. The Structure and Function of Mucins

Mucins are heavily glycosylated, high molecular weight (>200 kDa) transmembrane glycoproteins with one epidermal growth factor (EGF)-like domain and are coded by MUC genes [14]. They are produced by epithelial cells and classified by function and structure into two groups: transmembrane or secretory mucins. They are found throughout the body, including in the trachea and the upper GI tract [14]. 

Secretory mucins are coded by genes including MUC2, MUC5AC, MUC5B and MUC6 and produce a mucous gel, which protects and lubricates the underlying epithelial cells lining the upper GI tract [14,15,16,17,18,19]. In particular, a mucus-layer, predominantly composed of the MUC5AC mucin acts as a diffusion barrier, protecting underlying surface epithelial cells from luminal hydrochloric acid and subsequent erosion [17].

Transmembrane mucins, encoded by genes MUC1, MUC4, MUC13 and MUC16, contribute to this protective gel and are found in the epithelial cell membrane, allowing them to interact with cell surface molecules. They are key components of signal transduction in cell-to-cell signalling [14,15,16,17,18,19]. 

Mucins are extremely versatile and are involved in many complex pathophysiological processes. Their uses range from markers of disease severity to potential druggable targets to improve clinical outcomes, as well as biomarkers of early cancer [20,21,22,23]. An example of the former is the induction of MUC5AC during virus-induced exacerbation of chronic obstructive pulmonary disease, resulting in increased airway inflammation and severity of the exacerbation [24]. 

### 1.3. Mucins in Tumourigenesis

The scientific literature alludes to solid organ malignancies using mucins to promote tumourigenesis and metastasis by potentiating their functions in epithelial protection and intercellular signalling [21]. A proposed model is that immune effector cells produce inflammatory cytokines such as interleukin-6 (IL-6) and tumour necrosis factor alpha (TNF-α) that activate transcription factors including nuclear factor-κB (NF-κB), signal transducer and activator of transcription 1 (STAT1) and STAT3 in epithelial cells lining the GI tract [25,26,27]. Although MUC2 limits the inflammatory response at the apical membrane, and the activation of MUC1 is associated with the promotion of cell proliferation and survival, chronic inflammation and prolonged stimulation of these pathways result in epithelial cells becoming susceptible to the accumulation of genetic mutations leading towards tumourigenesis [26]. 

Chronic inflammatory stimulation may occur from smoking or alcohol consumption, demonstrating an association between carcinogens and altered mucin gene expression, leading to the development of cancer [25]. Aberrant mucin gene expression may occur as a result of epigenetic influences, resulting in exposure of the underlying epithelium, leading to epithelial injury by gastric acid or microorganisms, neoplasia and metastatic disease [26,27,28]. In the case of OC, this extends to the progression of the pre-malignant condition, Barrett’s metaplasia to OAc [18,29].

The scientific literature suggests an interplay between chronic inflammatory stimulation and epigenetic influence leading to aberrant mucin gene expression and eventually the development and progression of OGC. Examples of chronic inflammatory processes include exposure to environmental factors including smoke and pathogenic infections, which increase the chances of epigenetic modifications within healthy cells, eventually leading to tumourigenesis [30]. Modifications include DNA methylation and histone acetylation, resulting in the downregulation of tumour suppressor genes and promotion of oncogenes, contributing to the development of malignancy. Pro-inflammatory cytokines including interferon-γ and TNF-α correlate with the levels of methylation and together with reactive oxygen species (ROS) are released by tumour cells, driving the progression of cancer and metastasis through a positive feedback loop [30]. Epigenetic modifications triggered by inflammation, including site-specific DNA methylation and histone modification of mucin genes result in the silencing of tumour suppressor genes. Mucin genes including MUC2, MUC5AC and MUC6 are typically expressed in epithelial cells and are highly susceptible to the aforementioned modifications, leading to a greater risk of malignant transformation to OGC [31]. 

### 1.4. Methodologies in the Analysis of Mucin Expression in Oesophago-Gastric Tissue

Mucin expression in oesophago-gastric biopsies or surgically resected specimens may be elucidated by reverse transcriptase-polymerase chain reaction (RT-PCR) to detect mucin RNA or in situ hybridisation and immunohistochemical staining after interrogation with mouse monoclonal antibodies to quantify the expression of mucin core polypeptides [32,33,34].

Mucins have been implicated in the diagnosis, metastatic spread and recurrence of OGC [18,22,25,28,35]. The included studies describe ways in which mucins and their subcomponents, their epitopes and core polypeptides, can be used as the markers of progression from pre-malignant lesions to cancer, disease severity as well as the indicators of invasion and metastases. 

The aim of this systematic literature review is to define the role of mucins in the diagnosis of OGC, in particular, to highlight which mucins may be implicated in OGC. 

## 2. Materials and Methods 

### 2.1. Search Strategy

This systematic literature review was conducted in accordance with the Preferred Reporting Items for Systematic Reviews and Meta-Analysis (PRISMA) and Synthesis without Meta-Analysis protocols in observational studies and randomized trials [36,37]. It was registered in the international prospective register of systematic reviews (PROSPERO); registration number CRD42022324756. 

**Figure 1:**
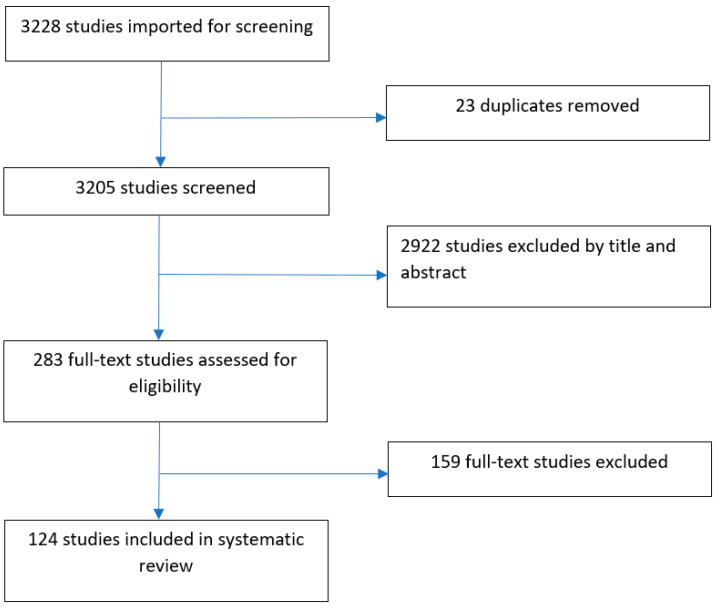
PRISMA flow diagram of included studies.

A literature search of Medline (PubMed), Web of Science, Embase and Cochrane databases was carried out by two co-authors, N.P. and G.G., on 1 July 2023 to capture studies published between January 1960 and December 2022. The following Medical Subject Headings (MeSH)’s terms were included in the search string: ‘mucin,’ ‘oesophageal cancer,’ ‘gastric cancer’ and ‘diagnosis’. Inclusion and exclusion criteria are shown in Table 1, and the total number of included studies is shown in Figure 1.

### 2.2. Data Extraction

**Figure 2:**
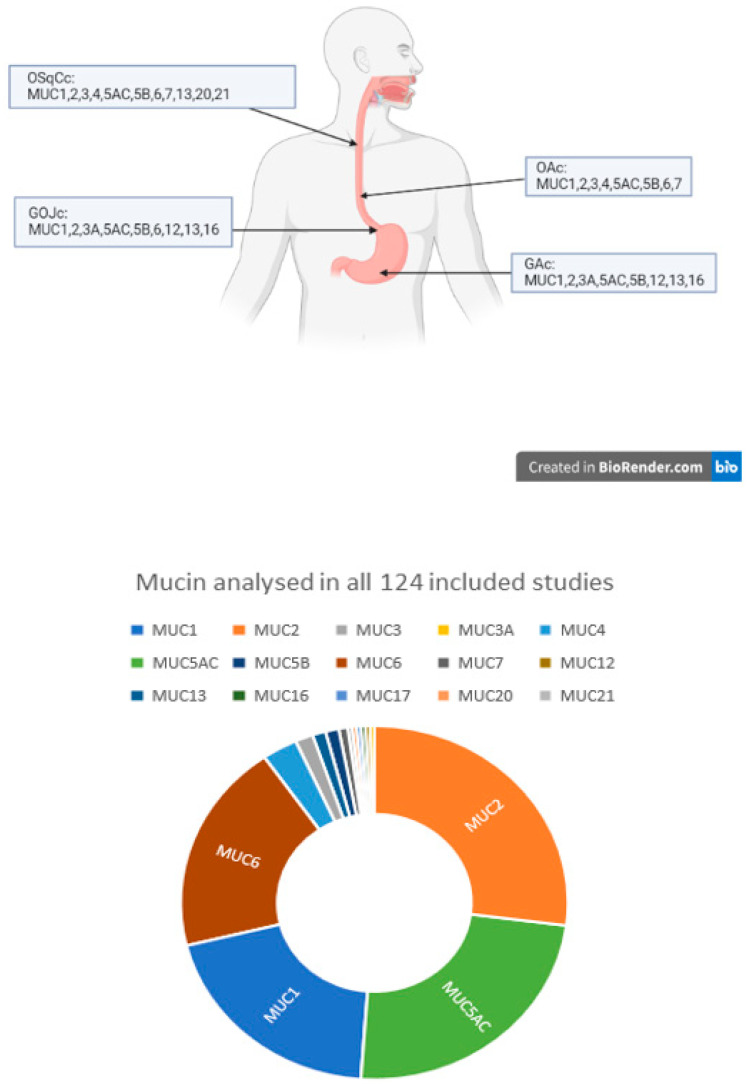
Mucins associated with each type of cancer as described in the included studies (OSqCc—oesophageal squamous cell carcinoma; OAc—oesophageal adenocarcinoma; GOJc—gastro-oesophageal junction cancer; GAc—gastric adenocarcinoma) and doughnut chart of mucins analysed in all included studies.

Two reviewers (N.P. and G.G.) independently screened titles and abstracts. Any conflicts were resolved following a discussion over the relevant study or with a senior reviewer if necessary. Studies eligible for inclusion were read in full before data relevant for the systematic review were extracted with the use of a data collection table. A PRISMA flow diagram is presented in Figure 1. Demographic components of included studies and the number of studies analysing each type of cancer and mucin are presented in Table 2 and Figure 2.

### 2.3. Study Quality and Risk of Bias Assessments

**Figure 3:**
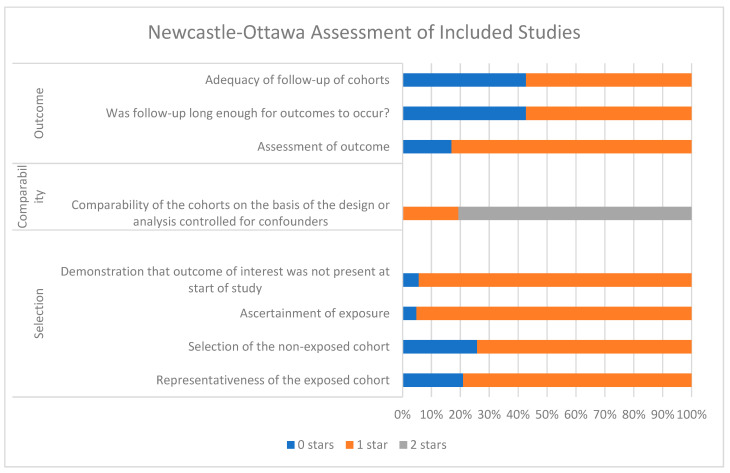
Newcastle–Ottawa assessment of included cohort studies.

The modified Newcastle–Ottawa scale for non-randomised cohort studies was used by two co-authors (N.P. and G.G.) to assess study quality and risk of bias, the results of which are summarized in Figure 3 and Supplementary Table S1 [38]. Each component of this scale is scored from 0 to 2 stars, allowing for a semi-quantitative assessment of study quality in which the level of bias in cohort selection, study design and assessment of outcomes for each included study may be rated. Two stars represent a low level of bias, and 0 stars represents a high level of bias. In this scale, the term ‘bias’ relates to how representative the data of each study are of wider, more diverse populations, and, therefore, how applicable the data are on a larger scale. The less representative and the less applicable, the higher the level of bias, and the lower the quality of the study as indicated by the scale.

### 2.4. Data Availability

Data were extracted from each of the included studies and analysed for the purposes of this systematic literature review. Evidence of the demographics of each study was collated. These included sampling techniques, mucins studied, analysis technique as well as a summary of the findings of each study. A subgroup analysis of each of the 3 types of malignancy was carried out and discussed individually.

## 3. Results

The initial literature search identified 3228 studies. After screening and assessment for full-text eligibility, 124 studies reporting 13,840 cancer specimens were included. The majority of studies were clinicopathologic and molecular analyses (87%), followed by cohort (9%), clinicopathologic and gene studies (2%) and population-based case–control studies (2%). The median age of patients was 63 years, and the most commonly used method of sampling was via surgical resection (72%) followed by endoscopic biopsy (16%). 

The most frequently analysed mucin was MUC2, investigated in 73 out of 124 studies (59%) followed by MUC5AC in 65 studies (52%), MUC1 in 54 studies (44%) and MUC6 in 52 studies (42%).

The most analysed pathology was gastric adenocarcinoma (GAc), in 101 out of 124 studies (81%) followed by oesophageal adenocarcinoma (OAc) in 24 studies (19%). Within OAc, four studies (3%) specified the investigation of adenocarcinoma of the gastro-oesophageal junction (GOJc). 

## 4. Discussion

Underpinning this systematic literature review is the importance of early diagnosis in improving survival in oesophago-gastric cancer (OGC). We addressed this by exploring whether mucins have a role in enabling early diagnosis; we reviewed the most commonly analysed mucins, sampling and analysis techniques and the correlation between the mucin expression and the stage of disease.

### 4.1. Oesophageal Squamous Cell Carcinoma

A total of 10 studies included the analysis of mucins in oesophageal squamous cell carcinoma (OSqCc). All specimens were obtained by surgical resection and, MUC1 was the most commonly analysed mucin (8 out of 10 studies). Guillem et al. identified that all squamous cell epithelium, despite not being mucus-secreting, expresses both MUC1 and MUC4 and maintains this in OSqCc [35].

The expression of MUC1 has been linked to cancer invasion and metastasis, as well as being seen as a biomarker that can distinguish between pre-cancerous and healthy tissue [39,40,41,42]. Immunohistochemistry and real-time PCR were used to identify MUC1 expression at the protein and mRNA level, respectively. Although the upregulation of MUC1 in the primary tumour correlates with metastatic recurrence after surgical resection (*p* < 0.01), there has been little research into the use of MUC1 expression alone in the diagnosis of OSqCc [39]. In comparison, three studies commented on the use of MUC1 gene expression in the primary tumour as a predictor of cancer metastasis, and one study on the presence of MUC1 as a predictor of lymph node (LN) recurrence [40,41,42]. The proposed mechanism implies that MUC1 can be expressed on the entire cell surface and reduces both E-Cadherin-mediated cell-to-cell adhesion through steric hindrance and integrin-mediated cell adhesion through the extracellular matrix, thereby promoting systemic disease spread [40,42].

Furthermore, Song et al. identified significant overexpression of MUC1 in the cases of OSqCc with LN metastases, in comparison to those without metastases [38]. Their study identified that high expression of MUC1, classified as >50% of neoplastic cells with positive immunostaining, is related to poorer prognosis of OSqCc compared to low levels of MUC1 expression (0 to 50% positive staining) (*p* < 0.05). The expression of MUC1 protein in OSqCc specimens with metastasis was also higher than those without metastasis (*p* < 0.05). Additionally, three studies identified a positive correlation between the levels of MUC1 expression and the degree of LN metastases [39,40,42]. This may suggest that high MUC1 expression may have a role as an indicator of risk of LN metastases, which is associated with poorer outcomes in oesophageal carcinoma [5,42]. 

In comparison, one study reported that the MUC4 gene was expressed in all stages of OSqCc differentiation [35]. Guillem et al. investigated MUC gene expression in normal and pre-malignant oesophageal mucosa from 40 surgical specimens in patients undergoing oesophagectomy for Barrett’s oesophagus with OAc, or for OSqCc, and identified that MUC1 and MUC4 were the most frequently expressed mucin genes [35]. In particular, MUC4 was expressed more intensely than MUC1 in superficial epithelium based on signals obtained via in situ hybridisation. However, both mucin genes were expressed in normal mucosa and OSqCc, which suggests that alone they could not be used as diagnostic biomarkers or in risk stratification for OSqCc. Therefore, analyses such as ELISA or antibody detection-based Western blot could be used to quantify the differences in the presence of MUC4 mucin in healthy and cancerous oesophageal tissue. However, ELISA and Western blot are limited in terms of clinical translation into diagnostic tests. Therefore, novel assays must be developed for clinical application before MUC4 could be used as a possible diagnostic biomarker of oesophageal cancer.

In order to develop an effective risk stratification test, patient acceptability of the test is an important factor for consideration. Biological samples that can be obtained through non-invasive means such as saliva, could be targeted to identify biomarkers indicative of neoplasia. Oesophageal mucus, secreted by submucosal glands contains MUC5B mucins, which, importantly, have also been found in salivary mucus [35]. This is attributed to the fact that the upper aerodigestive tract arises from the common development of the primitive foregut [35,43]. Guillem et al. confirmed this in healthy, control oesophageal mucosa but not in OSqCc [35]. This study is limited in that the presence of MUC5B mucin in healthy mucosa was not quantified in terms of the protein expression, and the pathway by which MUC5B gene expression could be downregulated in the development of OSqCc is unknown. This presents an avenue for further research in quantifying the presence of the MUC5B mucin in salivary mucus of healthy control subjects and patients with OSqCc, in the pursuit of a non-invasive risk stratification test to enable early diagnosis of OGC. 

MUC1 expression in OSqCc upregulates the expression of matrix metalloproteinase-13 (MMP-13); this protein is highly expressed in patients with metastatic OSqCc and is indirectly regulated by the NF-kB pathway, which further supports the notion that MUC1 expression may be associated with advanced disease [40,41,42]. 

In summary, the literature highlights that MUC1 and MUC4 are the most frequently reported mucin genes associated with OSqCc. However, the included studies reported contrasting readouts, ranging from mucin gene expression to the presence of mucin at the protein level. There are no quantifiable threshold levels at which the MUC1 or MUC4 proteins are indicative of malignancy based on the findings of this systematic literature review. Although they may not be able to be used in isolation as a diagnostic biomarker, they may have a role in detecting more advanced disease and possible use in surveillance after potentially curative treatment. 

### 4.2. Oesophageal Adenocarcinoma

The precursor lesion to OAc is Barrett’s oesophagus (BO); this is a metaplastic change from squamous to columnar epithelium as a result of gastro-oesophageal reflux and carries an incidence of 1% in the UK [44,45]. Currently, patients undergo surveillance by oesophago-gastro-duodenoscopy (OGD) due to the 1% annual risk of progression to OAc. However, this is an invasive and uncomfortable procedure, and there is insufficient evidence to suggest that this is cost-effective [46,47]. Although OGD is the gold-standard test for upper gastrointestinal (GI) symptoms, 56% of OGDs performed each year in the UK are deemed ‘inappropriate’ [46,47,48]. This reinforces the importance of risk stratification to better select patients, who are susceptible to OAc or at risk of progression to malignancy. 

A total of 20 studies investigated mucin gene expression in oesophageal adenocarcinoma (OAc), analysing 662 tumour samples obtained by surgical resection, endoscopic biopsy or endoscopic mucosal resection. MUC1 and MUC2 were the most frequently analysed mucin genes; both were investigated in 12/20 studies, followed by MUC5AC in 11/20 studies. 

Piessen et al. identified that bile acid, which is a major component of gastro-oesophageal reflux and a tumour promoter, upregulates MUC4 expression [29,47]. Specifically, hepatocyte nuclear factor (HNF) 1α and HNFα transcription factors potentiate the bile acid upregulation of MUC4 and, consequently, the expression of MUC4 increases in oesophageal metaplasia and adenocarcinoma [29]. It has been suggested to be a potential early diagnostic tumour marker as it is expressed in both high-grade dysplasia as well as OAc [29,35].

It may be hypothesised that neoplasia is represented by a variety of changes in the expression of several mucin genes, instead of changes in the expression of a specific gene. In support of this, Burjonrappa et al. aimed to identify the change in mucin gene expression in the progression from BO to dysplasia and then OAc to aid in early diagnosis [14]. Their study identified that the expression of the MUC1 gene increases in the progression from healthy oesophageal mucosa to adenocarcinoma, whereas the expression of secretory mucin genes such as MUC2 decreases in progression to OAc, resulting in loss of protection from gastric acid and subsequently reduced mucosal repair [14]. With regard to an increase in MUC1 gene expression, this is echoed by four other studies in this subgroup analysis [2,25,27,28]. Similar behaviour of MUC1 was identified by Song et al. in the cases of OSqCc; greater MUC1 expression is associated with more advanced disease [42]. Thus, further work can focus on identifying a profile of changes in MUC gene expression in the development of oesophageal neoplasia.

In a study of 52 specimens by Piessen et al., strong expression of MUC1 as well as MUC4 in Barrett’s-associated OAc was identified [47]. However, where an association has been made between increased MUC1 expression and advanced cancer with LN metastases in studies on OSqCc, the same does not hold true according to the current evidence of MUC1 and MUC4 in OAc [47]. Furthermore, this study concluded that MUC1 and MUC4 cannot be used as diagnostic biomarkers to facilitate early detection of OAc. This study is limited by its statistical power in that only 52 specimens were analysed, which emphasises the need for a larger analysis profiling mucin expression throughout the upper GI tract, in healthy controls, patients with BO and established OAc [49]. At the level of the mucin protein, a quantitative assay such as ELISA could be used, whereas quantitative reverse transcription polymerase chain reaction (RT-qPCR) could be used for the detection and quantification of mucin mRNA. This may enable the diversity of different mucins in OG samples to be evaluated and compared between healthy, pre-malignant and cancerous samples. Differences in the mucins between these cohorts could be investigated further to identify potential biomarkers indicative of early disease.

A total of four studies looked specifically at the adenocarcinoma of the oesophago-gastric junction (GOJc), analysing MUC1, MUC2, MUC5AC and MUC6 in 191 surgically resected specimens [31,33,50,51]. All four studies were explicit in that they distinguished GOJc from distal oesophageal and proximal gastric cancers. In particular, Tajima et al. defined the GOJ as the junction between the end of the tubular oesophagus and the proximal heads of the gastric folds, and tumours arising from within the 4 cm segment of this were classified as GOJc [33]. 

Tajima et al. reported the protein expression of MUC6 in 67.3% and of MUC2 in 59.6% of the 52 cases of GOJc, and, although healthy mucosa was sampled alongside tumour, their study did not elaborate on MUC2 and MUC6 protein expression in noncancerous tissue [33]. Furthermore, this study identified that MUC2 expression decreases in the cases of advanced junctional cancers, which suggests that the MUC2 gene, similar to MUC1 in OSqCc, could be used as an indicator of earlier disease [2,23,31,41,50]. 

Of the 29 junctional adenocarcinomas analysed by Flucke et al., relative frequency of immunoreactivity for MUC1 was the highest compared to MUC2 and MUC5AC, indicating that of all the GOJ tumours staining positively, the MUC1 gene was expressed the most prominently in tumour tissue [23]. The mucin expression profile of GOJc was mixed, containing high MUC2 gene expression consistent with oesophageal and high MUC6 gene expression consistent with gastric cancers [51,52]. This suggests that there is no specific mucin gene profile for GOJc, which may make early identification of these tumours more challenging. This creates a potential avenue for further research to improve the understanding of the mucin gene expression in GOJc by stratifying tumours according to the Siewert classification. This is an anatomical classification, divided into types I-III depending on the epicentre of the tumour: distal oesophagus (type I), in the GOJ (type II) or within the first 5 cm of the stomach, extending towards the GEJ and oesophagus (type III).

Gulmann et al.’s study demonstrated that increased expression of MUC1 in junctional adenocarcinoma was associated with higher Tumour, Node, Metastasis (TNM) staging, which was also demonstrated by Sun et al. in OSqCc [40,53]. Quantification of the MUC1 protein expression in GOJc and OSqCc could be used to improve the risk stratification of patients and also identify those at potentially higher risk of recurrence. This could allow for more careful selection of patients, who may benefit from radical surgery and perioperative treatment, compared to those for whom non-surgical treatment could be more appropriate. 

### 4.3. Gastric Adenocarcinoma

A total of 101 of the included 124 studies investigated the mucin gene expression in gastric cancer (GC), substantially more than those in oesophageal cancer (34 studies). 

As with OAc, MUC1 was the most frequently analysed mucin (42/101 studies); however, other mucins including MUC2, MUC5AC and MUC6 appeared to be closely linked to the development of GC based on the findings of this systematic review [28,50,51,52,53,54].

In particular, Babu et al. found that the expression patterns of mucin genes MUC2, MUC5AC and MUC6 change in line with the development of intestinal metaplasia in Helicobacter pylori-infected gastric mucosa, during the progression to carcinoma [30]. The included studies on GC suggest that changes in the expression patterns can be triggered by various known aetiological factors including known carcinogens such as smoking, alcohol consumption and Helicobacter pylori infection, whereas the included studies on oesophageal and junctional cancer did not suggest any such influence [30]. 

These aetiologies may drive chronic inflammation, causing the impairment of the protective role of mucins in epithelial cells, thereby promoting tumourigenesis. However, Helicobacter pylori has also been implicated as an epigenetic aberration leading to the pathogenesis of GC by inducing extensive DNA methylation alterations in gastric epithelial cells [55,56]. In their study, Ge et al. reported that MUC1 is associated with the methylation of trefoil factor family 2 (TFF2), a secreted peptide that promotes epithelial repair, in Helicobacter pylori-infected gastric cells [55]. However, the mechanism through which this occurs is yet to be elucidated [57]. Furthermore, aberrant MUC1, following H. pylori infection regulates the methylation status of TFF2, thus contributing to the silencing of this peptide, leading towards GC development [57]. A further study by Shi et al. identified that the methylation of the promoter region in the MUC6 gene leads to the downregulation of MUC6 expression, which may promote the metastasis of GC [58]. This was corroborated by significantly lower levels of MUC6 expression in advanced and poorly differentiated gastric tumour specimens, than normal and pre-malignant tissue in their study (*p* < 0.01) [58].

As highlighted by Yamanoi and Nakayama, the expression of residues attached to mucin core proteins may provide further insight into biomarkers indicative of malignancy or greater risk of malignant transformation in OGC [59]. Their analysis of the *O*-glycans carrying terminal α1,4-linked *N*-acetylglucosamine (αGlcNAc) suggests that this is a potential tumour-suppressing molecule. Reduced αGlcNAc expression of MUC6, as determined by immunohistochemistry, occurs in chronic atrophic gastritis and pyloric gland adenoma, both of which are precursors to GAc. Additionally, reduced expression of αGlcNAc relative to MUC6 in Barrett’s oesophagus may be indicative of progression to OAc. This study indicates that mucin core proteins act as a scaffold upon which molecules which are the true drivers of tumourigenesis, may be anchored [59]. This warrants further investigation when attempting to identify the biomarkers of early malignant transformation in OGC.

From this subgroup analysis, there are discrepancies in the expression patterns of MUC2, MUC5AC and MUC6 in GC amongst the included studies. A number of studies commented on the involvement of these mucin genes in carcinogenesis, particularly that their expression increases consistently with the development of GC; however, other studies contradicted this. MUC2 expression decreases during metaplasia from normal gastric mucosa to intestinal-type, whereas MUC5AC is upregulated with more aggressive gastric tumours, and MUC6 is downregulated in gastric cancer [15,58,60,61,62]. Although the majority of studies (90/101) in this subgroup analysis used immunohistochemistry, there was significant variation in the antibodies for staining including human antibodies, NCL-HGM-45M1, as well as mouse-derived antibodies, NCL-MUC-1 and MCL-MUC-2, which may contribute to the heterogeneity in the results [15,50,60,61,62]. 

A number of studies have suggested that specific mucin gene expression patterns can be indicative of either intestinal or diffuse GC subtypes, and the expression patterns change over time [15,61,62,63,64]. Furthermore, studies have demonstrated variations in the mucin gene expression in the immediate vicinity of the tumour [15,53,60,61,62,63,64,65,66,67]. For example, Lee et al. reported complete the absence of MUC2 in normal gastric mucosa in patients with GC, followed by the expression of MUC2 in 97.8% of the areas of intestinal metaplasia in the stomach, followed by 55.4% positive expression of MUC2 in early GC [15]. The mechanism by which this occurs is not described in these studies but given its varying level of expression in gastric tumourigenesis, may be epigenetic in nature. 

Overall, only four of the included one hundred and twenty-four included studies specified whether the patients had undergone neoadjuvant therapy prior to sample collection. Therefore, it is unclear whether chemotherapy and radiotherapy influenced mucin gene expression and potentially affect the use of mucins in the diagnostic workup for OGC, as well as in the detection of advanced disease or its loco-regional recurrence. The previous literature has, however, established that neoadjuvant therapy modifies the single-cell transcriptional landscape of OGC, which may also be extended to include mucin expression [68,69]. Consequently, previous oncological treatment may represent a confounding variable that will need to be controlled for in further work on the role of mucins in OGC [69]. 

In addition to neoadjuvant therapy, diet is a major factor that influences mucin gene expression in the upper GI tract [70]. Digestive responses by the oesophagus and stomach to different foods modulate the contribution of mucin to endogenous protein components and their qualitative composition [70]. This may continue beyond the 6 h conventional fasting period patients are required to follow before upper GI endoscopy or surgery under general anaesthesia. As such, diet is an important variable which should be accounted for in further research on the mucin expression in OGC.

The majority of the included studies examined changes in the expression of mucin genes in OGC pathogenesis, as opposed to quantifying mucin protein in the samples. In terms of incorporating mucins into early diagnostic testing and risk stratification for OGC, quantifiable assays are needed to determine thresholds at which mucin proteins are indicative of malignancy. Future research should, therefore, take this into consideration. 

From the included studies, the challenge with using mucin gene expression in isolation as diagnostic biomarkers in OGC arises from the fact that these expression profiles also change in benign pathology including chronic gastritis and peptic ulcers [71]. Therefore, further research must be carried out to determine potential threshold levels of mucin gene expression, which are more indicative of early OGC, and not benign diseases such as gastro-oesophageal reflux disease (GORD). This will help with the risk stratification of patients for definitive investigations by way of endoscopy and biopsy to make a formal diagnosis of malignancy.

## 5. Conclusions 

A large number of studies have been carried out investigating the mucin profile of oesophago-gastric cancer (OGC). From our review, we can conclude that mucin gene expression is altered in the pathogenesis of OGC. However, the significant heterogeneity between the results of the included studies makes drawing more precise conclusions about the relationship between mucin gene expression and OGC limited. 

In particular, there is significant heterogeneity among the included studies. For example, there is inconsistency in how studies define a true gastro-oesophageal junction tumour. Secondly, certain studies in each subgroup analysis differ in terms of their findings, particularly in relation to whether certain mucin genes are upregulated or downregulated in carcinogenesis. Reasons for changes in gene expression may be epigenetic or mutational, and further research into the underlying mechanisms is needed. Furthermore, this raises the question as to whether particular mucin proteins can be used as a diagnostic biomarker for OGC. Although the majority of studies used similar techniques including immunohistochemistry and cytoplasmic staining, the different antibodies used in the staining process may account for some of the variation in the results yielded. 

MUC1, MUC2, MUC5AC and MUC6 were the most commonly analysed mucins across all three cancer types according to this literature review. These may form the basis of a larger cohort study, formally profiling mucin expression along the oesophagus and stomach at consistent anatomical locations in patients with and without treatment for early and advanced OGC and pre-malignant conditions including Barrett’s metaplasia, as well as healthy controls and those with a benign disease such as GORD. Such a study would be required to formally identify changes in mucin profile in oesophageal and gastric tumourigenesis that may have clinical applications.

## Figures and Tables

**Table 1:** Inclusion and exclusion criteria.

Inclusion Criteria	Exclusion Criteria
Studies on the role of MUC genes in the diagnosis of OGC: – Oesophageal adenocarcinoma (OAc); – Oesophageal squamous cell carcinoma (OSqCc); – Gastric adenocarcinoma (GAc); – Gastro-oesophageal junction adenocarcinoma (GOJAc).	Studies not assessing MUC gene expression in OGC diagnosis.
	Studies on secondary oesophageal or gastric cancers or metastases to the oesophagus or stomach from a non-OG primary cancer.
	Studies assessing the role of mucins in treatment response or prognosis of OGC.
	Paediatric cohorts (<16 years)
	Systematic literature reviews, meta-analyses
	Articles not published in English
	Case reports
	Case series of <10 patients
	Letters to the editor
	Conference abstracts

**Table 2:** Demographic details from included studies.

	*n*
**Total number of studies**	124
**Type of study**	
Cohort	11
Clinicopathologic and molecular analyses	108
Population-based case–control study	2
Clinicopathologic and gene study	3
**Females**	3801
**Males**	7585
**Median age** (Calculated from all median ages in included studies)	63.0 years
**Sampling technique** (9 studies used >1 sampling method)	
Endoscopic biopsy	21
Endoscopic mucosal resection/submucosal dissection	8
Surgical resection	96
Nasogastric aspirates	1
Not stated	7
**Type of cancer**	**Number of studies analysing cancer (*n*)**
Oesophageal adenocarcinoma	24
Oesophageal squamous cell carcinoma	10
Gastric adenocarcinoma	101

## Supplementary Materials

The following supporting information can be downloaded at: https://www.mdpi.com/article/10.3390/cancers15215252/s1. Table S1: Total Newcastle-Ottawa quality assessment scores for all included studies. References [72,73,74,75,76,77,78,79,80,81,82,83,84,85,86,87,88,89,90,91,92,93,94,95,96,97,98,99,100,101,102,103,104,105,106,107,108,109,110,111,112,113,114,115,116,117,118,119,120,121,122,123,124,125,126,127,128,129,130,131,132,133,134,135,136,137,138,139,140,141,142,143,144,145,146,147,148,149,150,151,152] are cited in the supplementary materials.

## References

[1] Patruni S., Fayyaz F., Bien J., Phillip T., King D.A. (2023). Immunotherapy in the Management of Oesophagogastric Cancer: A Practical Review. JCO Oncol. Pract..

[2] Butt M.A., Pye H., Haidry R.J., Oukrif D., Khan S.-U.-R., Puccio I., Gandy M., Reinert H.W., Bloom E., Rashid M. (2017). Upregulation of mucin glycoprotein MUC1 in the progression to oesophageal adenocarcinoma and therapeutic potential with a targeted photoactive antibody-drug conjugate. Oncotarget.

[3] Bray F., Ferlay J., Soerjomataram I., Siegel R.L., Torre L.A., Jemal A. (2018). Global Cancer Statistics 2018: GLOBOCAN Estimates of Incidence and Mortality Worldwide for 36 Cancers in 185 Countries. CA Cancer J. Clin..

[4] Park M.H., Wahedally M.A.H., Maynard N., Crosby T., Thomas B., Trudgill N., Geisler J., Napper R., Cromwell D. (2023). National Oesophago-Gastric Cancer Audit.

[5] Bird-Lieberman E.L., Fitzgerald R.C. (2009). Early diagnosis of oesophageal cancer. Br. J. Cancer.

[6] Rahman S., Thomas B., Maynard N., Park M.H., Wahedally M., Trudgill N., Crosby T., Cromwell D.A., Underwood T. (2022). J Impact of postoperative chemotherapy on survival for oesophagogastric adenocarcinoma after preoperative chemotherapy and surgery. Br. J. Surg..

[7] Arnal M.J.D., Arenas A.F., Arbeloa A.L. (2015). Esophageal cancer: Risk factors, screening and endoscopic treatment in Western and Eastern countries. World J. Gastroenterol..

[8] Machlowska J., Baj R., Sitarz M., Maciejewski R., Sitarz R. (2020). Gastric Cancer: Epidemiology, Risk Factors, Classification, Genomic Characteristics and Treatment Strategies. Int. J. Mol. Sci..

[9] Fitzgerald R.C., di Pietro M., O’Donovan M., Maroni R., Muldrew B., Debiram-Beecham I., Gehrung M., Offman J., Tripathi M., Smith S.G. (2020). Cytosponge-trefoil factor 3 versus usual care to identify Barrett’s oesophagus in a primary care setting: A multicentre, pragmatic, randomised controlled trial. Lancet.

[10] Ross-Innes C.S., Debiram-Beecham I., O’Donovan M., Walker E., Varghese S., Lao-Sirieix P., Lovt L., Griffin M., Ragunath K., Haidry R. (2015). Evaluation of a minimally invasive cell sampling device coupled with assessment of trefoil factor 3 expression for diagnosing Barrett’s oesophagus: A multi-center case-control study. PLoS Med..

[11] Huang J., Kumar S., Abbassi-Ghadi N., Spanĕl P., Smith D., Hanna G.B. (2013). Selected ion flow tube mass spectrometry analysis of volatile metabolites in urine headspace for the profiling of gastro-oesophageal cancer. Anal. Chem..

[12] Kumar S., Huang J., Abbassi-Ghadi N., Spanĕl P., Smith D., Hanna G.B. (2013). Selected ion flow tube mass spectrometry analysis of exhaled breath for volatile organic compound profiling of oesophago-gastric cancer. Anal. Chem..

[13] Kumar S., Huang J., Abbassi-Ghadi N., Mackenzie H.A., Veselkov K.A., Hoare J.M., Lovat L.B., Spanĕl P., Smith D., Hanna G.B. (2015). Mass Spectrometric Analysis of Exhaled Breath for the Identification of Volatile Organic Compound Biomarkers in Oesophageal and Gastric Adenocarcinoma. Ann. Surg..

[14] Burjonrappa S.C., Reddimasu S., Nawaz Z., Gao Z., Sharma P., Loggie B. (2007). Mucin expression profile in Barrett’s, dysplasia, adenocarcinoma sequence in the oesophagus. Indian J. Cancer.

[15] Lee H.-W., Yang D.H., Kim H.K., Lee B.H., Choi K.C., Choi Y.H., Park Y. (2007). E Expression of MUC2 in gastric carcinomas and background mucosae. J. Gastroenterol. Hepatol..

[16] Kufe D.W. (2009). Mucins in cancer: Function, prognosis and therapy. Nat. Rev. Cancer.

[17] Kwon J.-A., Lee S.-Y., Ahn E.-K., Seol S.-Y., Kim M.C., Kim S.J., Kim S.I., Chu I.-S., Leem S.-H. (2010). Short rare MUC6 minisatellites-5 alleles influence susceptibility to gastric carcinoma by regulating gene expression. Hum. Mutat..

[18] Niv Y., Ho S.B., Fass R., Rokkas T. (2018). Mucin Expression in the Oesophageal Malignant and Pre-malignant States: A Systematic Review and Meta-analysis. J. Clin. Gastroenterol..

[19] Hansson G.C. (2020). Mucins and the Microbiome. Annu. Rev. Biochem..

[20] Cui J., Yin Y., Ma Q., Wang G., Olman V., Zhang Y., Chou W.-C., Hong C.S., Zhang C., Cao S. (2015). Comprehensive characterization of the genomic alterations in human gastric cancer. Int. J. Cancer.

[21] Xiong Z.F., Shi J., Fu Z.H., Wan H.P., Tu L.X. (2017). Phenotype classification of gastric signet ring cell carcinoma and its relationship with K-ras mutation. Genet. Mol. Res..

[22] Toki F., Takahashi A., Aihara R., Ogata K., Ando H., Ohno T., Mochiki E., Kuwano H. (2010). Relationship between clinicopathological features and mucin phenotypes of advanced gastric adenocarcinoma. World J. Gastroenterol..

[23] Flucke U., Steinborn E., Dries V., Mönig S.P., Schneider P.M., Thiele J., Hölscher A.H., Dienes H.P., Baldus S.E. (2003). Immunoreactivity of cytokeratins (CK7, CK20) and mucin peptide core antigens (MUC1, MUC2, MUC5AC) in adenocarcinomas, normal and metaplastic tissues of the distal oesophagus, oesophago-gastric junction and proximal stomach. Histopathology.

[24] Singanayagam A., Footitt J., Marcynski M., Radicioni G., Cross M.T., Finney L.J., Trujillo-Torralbo M.-B., Calderazzo M., Zhu J., Aniscenko J. (2022). Airway mucins promote immunopathology in virus-exacerbated chronic obstructive pulmonary disease. J. Clin. Investig..

[25] Palmer A.J., Lochhead P., Hold G.L., Rabkin C.S., Chow W.-H., Lossowska J., Vaughan T.L., Berry S., Gammon M., Risch H. (2012). Genetic variation in C20orf54, PLCE1 and MUC1 and risk of upper gastrointestinal cancers in Caucasian populations. Eur. J. Cancer Prev..

[26] Cascio S., Zhang L., Finn O.J. (2011). MUC1 protein expression in tumor cells regulates transcription of proinflammatory cytokines by forming a complex with NF-κB p65 and binding to cytokine promoters: Importance of the extracellular domain. J. Biol. Chem..

[27] Benjamin J.B.E., Jayanthi V., Devaraj H. (2010). MUC1 expression and its association with other aetiological factors and localization to mitochondria in preneoplastic and neoplastic gastric tissues. Clin. Chim Acta.

[28] Yang J. (2020). Identification of novel biomarkers, MUC5AC, MUC1, KRT7, GAPDH, CD44 for gastric cancer. Med. Oncol..

[29] Piessen G., Jonckheere N., Vincent A., Hémon B., Ducourouble M.-P., Copin M.-C., Mariette C., Van Seuningen I. (2007). Regulation of the human mucin MUC4 by taurodeoxycholic and taurochenodeoxycholic bile acids in oesophageal cancer cells is mediated by hepatocyte nuclear factor 1α. Biochem. J..

[30] Babu S.D., Jayanthi V., Devaraj N., Reis C.A., Devaraj H. (2006). Expression profile of mucins (MUC2, MUC5AC and MUC6) in Helicobacter pylori infected pre-neoplastic and neoplastic human gastric epithelium. Mol. Cancer.

[31] Tajima Y., Yamazaki K., Makino R., Nishino N., Masuda Y., Aoki S., Kato M., Morohara K., Kusano M. (2007). Differences in the histological findings, phenotypic marker expressions and genetic alterations between adenocarcinoma of the gastric cardia and distal stomach. Br. J. Cancer.

[32] Rachagani S., Torres M.P., Moniaux N., Batra S.K. (2009). Current status of mucins in the diagnosis and therapy of cancer. Biofactors.

[33] Guillem P., Billeret V., Bisine M.P., Flejou J.F., Lecomte-Houcke M., Degand P., Aubert J.P., Triboulet J.P., Porchet N. (2000). Mucin gene expression and cell differentiation in human normal, premalignant and malignant oesophagus. Int. J. Cancer.

[34] Moher D., Liberati A., Tetzlaff J., Altman D.G., PRISMA Group (2010). Preferred reporting items for systematic reviews and meta-analyses: The PRISMA statement. Int. J. Surg..

[35] Campbell M., McKenzie J.E., Sowden A., Katikireddi S.V., Brennan S.E., Ellis S., Hartmann-Boyce J., Ryan R., Shepperd S., Thomas J. (2020). Synthesis without meta-analysis (SWiM) in systematic reviews: Reporting guideline. BMJ.

[36] The Newcastle-Ottawa Scale (NOS) for Assessing the Quality of Nonrandomised Studies in Meta-Analyses. Vol. Ottawa, Ontario, Canada: Community Medicine, University of Ottawa. http://www.ohri.ca/programs/clinical_epidemiology/oxford.htm.

[37] Sun Z.-G., Yu L., Yang F., Gao W., Wang Z., Zhu L.-M. (2016). Mucin 1 expression correlates with metastatic recurrence in postoperative patients with oesophageal squamous cell cancer. Pol. J. Pathol..

[38] Sun Z.-G., Yu L., Gao W., Wang Z., Zhu L.-M. (2018). Clinical and prognostic significance of MUC1 expression in patients with oesophageal squamous cell carcinoma after radical resection. Saudi J. Gastroenterol..

[39] Wang Y., Liao X., Ye Q., Huang L. (2018). Clinic implication of MUC1 0-glycosylation and C1GALT1 in oesophagus squamous cell carcinoma. Sci. China Life Sci..

[40] Song Z.-B., Gao S.-S., Yi X.-N., Li Y.-J., Wang Q.-M., Zhuang Z.-H. (2003). Expression of MUC1 in oesophageal squamous-cell carcinoma and its relationship with prognosis of patients from Linzhou city, a high incidence area of northern China. World J. Gastroenterol..

[41] Audie J.P., Janin A., Porchet N., Copin M.C., Gosselin B., Aubert J.P. (1993). Expression of human mucin genes in respiratory, digestive, and reproductive tracts ascertained by in situ hybridization. J. Histochem. Cytochem..

[42] Fitzgerald R.C., di Pietro M., Ragunath K., Ang Y., Kang J.-Y., Watson P., Trudgill N., Patel P., Kaye P.V., Sanders S. (2013). British Society of Gastroenterology guidelines on the diagnosis and management of Barrett’s oesophagus. Gut.

[43] de Jonge P.J.F., van Blankenstein M., Grady W.M., Kuipers E.J. (2014). Barrett’s oesophagus: Epidemiology, cancer risk and implications for management. Gut.

[44] Beg S., Ragunath K., Wyman A., Banks M., Trudgill N., Pritchard D.M., Riley S., Anderson J., Griffiths H., Bhandari P. (2017). Quality standards in upper gastrointestinal endoscopy: A position statement of the British Society of Gastroenterology (BSG) and Association of Upper Gastrointestinal Surgeons of Great Britain and Ireland (AUGIS). Gut.

[45] de Jong J.J., Lantinga M.A., Drenth J.P. (2019). Prevention of overuse: A view on upper gastrointestinal endoscopy. World J. Gastroenterol..

[46] O’Sullivan J.W., Albasri A., Nicholson B.D., Perera R., Aronson J.K., Roberts N., Heneghan C. (2018). Overtesting and undertesting in primary care: A systematic review and meta-analysis. BMJ Open.

[47] Piessen G., Wacrenier A., Briez N., Triboulet J.-P., Van Seuningen I., Mariette C. (2009). Clinical impact of MUC1 and MUC4 expression in Barrett-associated oesophageal adenocarcinoma. J. Clin. Pathol..

[48] Chinyama C.N., Marshall R.E., Owen W.J., Mason R.J., Kothari D., Wilkinson M.L., Sanderson J.D. (1999). Expression of MUC1 and MUC2 mucin gene products in Barrett’s metaplasia, dysplasia and adenocarcinoma: An immunopathological study with clinical correlation. Histopathology.

[49] Dwertmann Rico S., Mahnken M., Büscheck F., Dum D., Luebke A.M., Kluth M., Hube-Magg C., Hinsch A., Höflmayer D., Möller-Koop C. (2021). MUC5AC Expression in Various Tumor Types and Nonneoplastic Tissue: A Tissue Microarray Study on 10 399 Tissue Samples. Technol. Cancer Res. Treat..

[50] Takami H., Sentani K., Matsuda M., Oue N., Sakamoto N., Yasui W. (2012). Cytokeratin expression profiling in gastric carcinoma: Clinicopathologic significance and comparison with tumor-associated molecules. Pathobiology.

[51] Gulmann C., Counihan I., Grace A., Patchett S., Leen E., Leader M., Kay E. (2003). Cytokeratin 7/20 and mucin expression patterns in oesophageal, cardia and distal gastric adenocarcinomas. Histopathology.

[52] Choi J.S., Kim M.A., Lee H.E., Lee H.S., Kim W.H. (2009). Mucinous gastric carcinomas: Clinicopathologic and molecular analyses. Cancer.

[53] Guner G., Isik A., Karabulut E., Gedikoglu G., Sokmensuer C., Akyol A. (2018). Morphologic and Immunohistochemical Appraisal of Primary Gastric carcinomas. Appl. Immunohistochem. Mol. Morphol..

[54] Puyan F.O., Can N., Ozyilmaz F., Usta U., Sut N., Tastekin E., Altaner S. (2011). The relationship among PDX1, CDX2, and mucin profiles in gastric carcinomas; correlations with clinicopathologic parameters. J. Cancer Res. Clin. Oncol..

[55] Ge Y., Ma G., Liu H., Lin Y., Zhang G., Du M., Wang M., Chu H., Zhang H., Zhang Z. (2020). MUC1 is associated with TFF2 methylation in gastric cancer. Clin. Epigenetics.

[56] Ohba R., Iijima K. (2016). Pathogenesis and risk factors for gastric cancer after Helicobacter pylori eradication. World J. Gastrointest. Oncol..

[57] Qian Z., Jiang Y., Shou C., Yu J., Huang D., Xie H., Zhou L., Chen D., Zheng S. (2022). Validation of the DNA Methylation Lanscape of TFF1/TFF2 in Gastric Cancer. Cancers.

[58] Shi D., Xi X.-x. (2021). Regulation of MUC6 Methylation Correlates with Progression of Gastric Cancer. Yonsei Med. J..

[59] Yamanoi K., Nakayama J. (2018). Reduced αGlcNAc glycosylation on gastric gland mucin is a biomarker of malignant potential for gastric cancer, Barrett’s adenocarcinoma, and pancreatic cancer. Histochem. Cell Biol..

[60] Pinto-de-Sousa J., Reis C.A., David L., Pimenta A., Cardoso-de-Oliveira M. (2004). MUC5B expression in gastric carcinoma: Relationship with clinic-pathological parameters and with expression of mucins MUC1, MUC2, MUC5AC and MUC6. Virchows Arch..

[61] Kim D.H., Shin N., Kim G.H., Song G.A., Jeon T.-Y., Kim D.-H., Lauwers G.Y., Park D.Y. (2013). Mucin expression in gastric cancer: Reappraisal of its clinicopathologic and prognostic significance. Arch. Pathol. Lab. Med..

[62] Reis C.A., David L., Carvalho F., Mandel U., de Bolós C., Mirgorodskaya E., Clausen H., Sobrinho-Simões M. (2000). Immunohistochemical study of the expression of MUC6 mucin and co-expression of other secreted mucins (MUC5AC and MUC2) in human gastric carcinomas. J. Histochem. Cytochem..

[63] Javanbakht M., Akhavanmoghadam J., Talaei A.K., Aghyani M., Mozafari M., Khedmat L., Mohebbi M. (2017). Differential expression of two genes Oct-4 and MUC5AC associates with poor outcome in patients with gastric cancer. Clin. Exp. Pharmacol. Physiol..

[64] Li X.-H., Zheng H.-C., Wang Z.-G., Takahashi H., Yang X.-H., Guan Y.-F., Takano Y. (2008). The clinicopathological and prognostic significance of MUC-1 expression in Japanese gastric carcinomas: An immunohistochemical study of tissue microarrays. Anticancer. Res..

[65] Leteurtre E., Zerimech F., Piessen G., Wacrenier A., Leroy X., Copin M.-C., Mariette C., Aubert J.-P., Porchet N., Buisine M.-P. (2006). Relationships between mucinous gastric carcinoma, MUC2 expression and survival. World J. Gastroenterol..

[66] Sakamoto H., Yonezawa S., Utsunomiya T., Tanaka S., Kim Y.S., Sato E. (1997). Mucin antigen expression in gastric carcinomas of young and old adults. Hum. Pathol..

[67] Conze T., Carvalho A.S., Langegren U., Almeida R., Reis C.A., David L., Söderberg O. (2010). MUC2 mucin is a major carrier of the cancer-associated sialyl-Tn antigen in intestinal metaplasia and gastric carcinomas. Glycobiology.

[68] Myllykangas S., Junnila S., Kokkola A., Autio R., Scheinin I., Kiviluoto T., Karjalainen-Lindsberg M.-L., Hollmén J., Knuutila S., Puolakkainen P. (2008). Integrated gene copy number and expression microarray analysis of gastric cancer highlights potential target genes. Int. J. Cancer.

[69] Croft W., Evans R.P.T., Pearce H., Elshafie M., Griffiths E.A., Moss P. (2022). The single cell transcriptional landscape of oesophageal adenocarcinoma and its modulation by neoadjuvant chemotherapy. Mol. Cancer.

[70] Montagne L., Piel C., Lallès J.P. (2004). Effect of diet on mucin kinetics and composition: Nutrition and health implications. Nutr. Rev..

[71] Mitsuuchi M., Hinoda Y., Itoh F., Endo T., Satoh M., Xing P.X., Imai K. (1999). Expression of MUC2 gene in gastric regenerative, metaplastic, and neoplastic epithelia. J. Clin. Lab. Anal..

[72] Aihara R., Mochiki E., Nakabayashi T., Akazawa K., Asao T., Kuwano H. (2005). Clinical significance of mucin phenotype, beta-catenin and matrix metalloproteinase 7 in early undifferentiated gastric carcinoma. Br. J. Surg..

[73] Ando H., Aihara R., Ohno T., Ogata K., Mochiki E., Kuwano H. (2009). Prognostic significance of the expression of MUC1 and collagen type IV in advanced gastric carcinoma. Br. J. Surg..

[74] Boltin D., Gingold-Belfer R., Dickman R., Halpern M., Morgenstern S., Roth M., Layfer O., Vilkin A., Niv Y., Levi Z. (2014). Gastric mucin expression in first-degree relatives of gastric cancer patients. Eur. J. Gastroenterol. Hepatol..

[75] Chaves P., Cruz C., Dias Pereira A., Suspiro A., de Almeida J.C.M., Leitão Soares J. (2005). Gastric and intestinal differentiation in Barrett’s metaplasia and associated adenocarcinoma. Dis. Esophagus..

[76] Cheah P.-L., Ramachandran K. (1994). Alterations in mucin type: An indicator for suspicion of malignant gastric transformation. Malaysian J. Pathol..

[77] Chlumská A., Mukenšnabl P., Waloschek T., Zámečník M. (2018). Esophageal dysplasia and adenocarcinoma: A study with double immunostaining for intestinal and gastric markers. Cesk Patol..

[78] Cho K.J., Myong N.H., Jang J.J. (1991). Mucin histochemistry by paradoxical concanavalin A staining in early gastric carcinomas. J. Korean Med. Sci..

[79] Cui S.-J., Li Y., Zhou R.-M., Liu L., Cao S.-R., Huang X., Huo X.-R., Wang N. (2021). TIM-3 polymorphism is involved in the progression of esophageal squamous cell carcinoma by regulating gene expression. Environ. Mol. Mutagen..

[80] DiMaio M.A., Kwok S., Montgomery K.D., Lowe A.W., Pai R.K. (2012). Immunohistochemical Panel for Distinguishing Esophageal Adenocarcinoma from Squamous Cell Carcinoma: A Combination of p63, Cytokeratin 5/6, MUC5AC, and AGR2 Allows Optimal Subtyping. Hum. Pathol..

[81] Forné M., Fernández-Baňares F., González-Mínguez C., Casalots J., Garcia-Gil L.J., Esteve M., Esteve M., Rosinach M., Espinós J., Loras C. (2009). Lack of Clinical Usefulness of Das-1 Monoclonal Antibody and Mucin Expression as Risk Markers of Gastric Carcinoma in Patients With Gastric Intestinal Metaplasia. Am. J. Clin. Pathol..

[82] Fujita Y., Uesugi N., Sugimoto R., Eizuka M., Toya Y., Akasaka R., Matsumoto TSugai T. (2020). Analysis of clinicopathological and molecular features of crawling-type gastric adenocarcinoma. Diagn Pathol..

[83] Gűrbűz Y., Kahlke V., Klőppel G. (2002). How do gastric carcinoma classification systems relate to mucin expression patterns? An immunohistochemical analysis in a series of advanced gastric carcinomas. Virchows Arch..

[84] Han H.S., Lee S.-Y., Lee K.Y., Hong S.N., Kim J.H., Sung I.-K., Park H.S., Jin C.J., Min Y.I. (2009). Unclassified mucin phenotype of gastric adenocarcinoma exhibits the highest invasiveness. J. Gastroenterol. Hepatol..

[85] He L., Qu L., Wei L., Chen Y., Suo J. (2017). Reduction of miR-132-3p contributes to gastric cancer proliferation by targeting MUC13. Mol. Med. Rep..

[86] Jia Y., Persson C., Hou L., Zheng Z., Yeager M., Lissowska J., Chanock S.J., Chow W.-H., Ye W. (2010). A comprehensive analysis of common genetic variation in MUC1, MUC5AC, MUC6 genes and risk of stomach cancer. Cancer Causes Control..

[87] Kageyama-Yahara N., Yamamichi N., Takahashi Y., Nakayama C., Shiogama K., Inada K.-I., Konno-Shimizu M., Kodashima S., Fujishiro M., Tsutsumi Y. (2014). Gli regulates MUC5AC transcription in human gastrointestinal cells. PLoS ONE.

[88] Khor T.S., Alfaro E.E., Ooi E.M.M., Li Y., Srivastava A., Fujita H., Park Y., Kumarasinghe M.P., Lauwers G.Y. (2012). Divergent expression of MUC5AC, MUC6, MUC2, CD10, and CDX-2 in dysplasia and intramucosal adenocarcinomas with intestinal and foveolar morphology: Is this evidence of distinct gastric and intestinal pathways to carcinogenesis in Barrett Esophagus?. Am. J. Surg. Pathol..

[89] Li H., Yu B., Li J., Su L., Yan M., Zhang J., Li C., Zhu Z., Liu B. (2015). Characterization of Differentially Expressed Genes Involved in Pathways Associated with Gastric Cancer. PLoS ONE.

[90] Machado J.C., Nogueira A.M.M.F., Carneiro F., Reis C.A., Sobrinho-Simŏes M. (2000). Gastric carcinoma exhibits distinct types of cell differentiation: An immunohistochemical study of trefoil peptides (TFF1 and TFF2) and mucins (MUC1, MUC2, MUC5AC, and MUC6). J. Pathol..

[91] Mall A., McConney Z., Lotz Z., Tyler M., McLeod H., Hickman R., Dent D., Kahn D. (2000). Increased fragmentation of MUC 5AC mucins in gastric juice of patients with ulceration and carcinoma. S. Afr. J. Sci..

[92] Nakajima K., Ota H., Zhang M.X., Sano K., Honda T., Ishii K., Nakayama J. (2003). Expression of gastric gland mucous cell-type mucin in normal and neoplastic human tissues. J. Histochem. Cytochem..

[93] Ozcan H.E.A., Anuk T., Ozden O. (2018). Expression profile and cellular localizations of mucin proteins, CK7, and cytoplasmic p27 in Barrett’s esophagus and esophageal adenocarcinoma. Adv. Med. Sci..

[94] Reis C.A., David L., Seixas M., Burchell J., Sobrinho-Simŏes M. (1998). Expression of fully and under-glycosylated forms of MUC1 mucin in gastric carcinoma. Int. J. Cancer.

[95] Song K., Yang Q., Yan Y., Yu X., Xu K., Xu J. (2021). Gastric mucin phenotype indicates aggressive biological behaviour in early differentiated gastric adenocarcinomas following endoscopic treatment. Diagn Pathol..

[96] Sugai T., Habano W., Uesugi N., Jao Y.-F., Nakamura S-i Abe K., Takagane A., Terashima M. (2004). Three independent genetic profiles based on mucin expression in early differentiated-types gastric cancers—A new concept of genetic carcinogenesis of early differentiated-type adenocarcinomas. Mod Pathol..

[97] Tamura Y., Higashi M., Kitamoto S., Yokoyama S., Osako M., Horinouchi M., Shimizu T., Tabata M., Batra S.K., Goto M. (2012). MUC4 and MUC1 expression in adenocarcinoma of the stomach correlates with vessel invasion and lymph node metastasis: An immunohistochemical study of early gastric cancer. PLoS ONE.

[98] Tsukashita S., Kushima R., Bamba M., Sugihara H., Hattori T. (2001). MUC gene expression and histogenesis of adenocarcinoma of the stomach. Int. J. Cancer.

[99] Wang R.-Q., Fang D.-C. (2003). Alterations of MUC1 and MUC3 expression in gastric carcinoma: Relevance to patient clinicopathological features. J. Clin. Pathol..

[100] Wang K., Yuen S.T., Xu J., Lee S.P., Yan H.H.N., Shi S.T., Siu H.C., Deng S., Chu K.M., Law S. (2014). Whole-genome sequencing and comprehensive molecular profiling identify new driver mutations in gastric cancer. Nat. Genet..

[101] Yamada S., Yamanoi K., Sato Y., Nakayama J. (2020). Diffuse MIST1 expression and decreased α1,4-linked N-acetylglucosamine (αGlcNAc) glycosylation on MUC6 are distinct hallmarks for gastric neoplasms showing oxyntic gland differentiation. Histopathology.

[102] Endo T., Tamaki K., Arimura Y., Itoh F., Hinoda Y., Hareyama M., Irimura T., Fujita M., Imai K. (1998). Expression of sulphated carbohydrate chain and core peptides of mucin detected by monoclonal antibodies in Barrett’s esophagus and esophageal adenocarcinoma. J. Gastroenterol..

[103] Higuchi K., Nishikura K., Ajioka Y., Watanabe G. (2006). Macroscopic Findings and Mucous Phenotypes of Early Gastric Depressed Type Carcinomas. Acta Med. Biol..

[104] Yu T., Chen X., Lin T., Liu J., Li M., Zhang W., Xu X., Zhao W., Liu M., Napier D.L. (2016). KLF4 deletion alters gastric cell lineage and induces MUC2 expression. Cell Death Dis..

[105] Tian Y., Denda-Nagai K., Kamata-Sakurai M., Nakamori S., Tsukui T., Itoh Y., Okada K., Yi Y., Irimura T. (2012). Mucin 21 in esophageal squamous epithelia and carcinomas: Analysis with glycoform-specific monoclonal antibodies. Glycobiology.

[106] Setia N., Agoston A.T., Han H.S., Mullen J.T., Duda D.G., Clark J.W., Deshpande V., Mino-Kenudson M., Srivastava A., Lennerz J.K. (2016). A protein and mRNA expression-based classification of gastric cancer. Mod. Pathol..

[107] Gűrbűz Y., Klőppel G. (2004). Differentiation pathways in duodenal and ampullary carcinomas: A comparative study on mucin and trefoil peptide expression, including gastric and colon carcinomas. Virchows Arch..

[108] Wang Y.Z., Mitomi H., Kurihara M., Ishihara K., Hotta K., Tanigawa Okayasu I. (2000). Gastric adenomas and superficial adenocarcinomas display distinct patterns of mucin carbohydrate and core protein expression. Histopathology.

[109] Semino-Mora C., Doi S.Q., Marty A., Simko V., Carlstedt I., Dubois A. (2003). Intracellular and interstitial expression of Helicobacter pylori virulence genes in gastric precancerous intestinal metaplasia and adenocarcinoma. J. Infect. Dis..

[110] Fan X.-N., Karsten U., Goletz S., Cao Y. (2010). Reactivity of a humanized antibody (hPankoMab) towards a tumor-related MUC1 epitope (TA-MUC1) with various human carcinomas. Pathol. Res. Pract..

[111] Buisine M.P., Devisme L., Maunoury V., Deschodt E., Gosselin B., Copin M.C., Aubert J.P., Porchet N. (2000). Developmental mucin gene expression in the gastroduodenal tract and accessory digestive glands. I. Stomach. A relationship to gastric carcinoma. J. Histochem. Cytochem..

[112] Weimann A., Rieger A., Zimmermann M., Gross M., Hoffmann P., Slevogt H., Morawietz L. (2010). Comparison of six immunohistochemical markers for the histologica diagnosis of neoplasia in Barrett’s esophagus. Virchows Arch..

[113] Begnami M.D., Fregnani J.H.T.G., Brentani H., Torres C., Costa W.L., Montagnini A., Nonogaki S., Soares F.A. (2011). Identification of protein expression signatures in gastric carcinomas using clustering analysis. J. Gastroenterol. Hepatol..

[114] Ushiku T., Arnason T., Ban S., Hishima T., Shimizu M., Fukayama M., Lauwers G.Y. (2013). Very well-differentiated gastric carcinoma of intestinal type: Analysis of diagnostic criteria. Mod. Pathol..

[115] Szachnowicz S., Cecconello I., Ribeiro U., Iriya K., El Ibrahim R., Takeda F.R., Corbett C.E.P., Safatle-Ribeiro A.V. (2009). Mucin pattern reflects the origin of the adenocarcinoma in Barrett’s esophagus: A retrospective clinical and laboratorial study. World J. Surg. Oncol..

[116] Zhang H.-K., Zhang Q.-M., Zhao T.-H., Li Y.-Y., Yi Y.-F. (2004). Expression of mucins and E-cadherin in gastric carcinoma and their clinical significance. World J. Gastroenterol..

[117] Wang J.Y., Chang C.T., Hsieh J.S., Lee L.W., Huang T.J., Chai C.Y., Lin S.R. (2003). Role of MUC1 and MUC5AC expressions as prognostic indicators in gastric carcinomas. J. Surg. Oncol..

[118] Kang H., An H.J., Song J.Y., Kim T.H., Heo J.H., Ahn D.H., Kim G. (2012). Notch3 and Jagged2 contribute to gastric cancer development and to glandular differentiation associated with MUC2 and MUC5AC expression. Histopathology.

[119] Fujimoto A., Ishikawa Y., Ishii T., Yamada A., Igarashi Y., Ohmoto Y., Kaise M. (2017). Differences between gastric signet-ring cell carcinoma and poorly differentiated adenocarcinoma: A comparison of histopathologic features determined by mucin core protein and trefoil factor family peptide immunohistochemistry. Pathol. Int..

[120] Ide M., Kato T., Ogata K., Mochiki E., Kuwano H., Oyama T. (2012). Keratin 17 expression correlates with tumor progression and poor prognosis in gastric adenocarcinoma. Ann. Surg. Oncol..

[121] Streppel M.M., Vincent A., Mukherjee R., Campbell N.R., Chen S.H., Konstantopoulos K., Goggins M.G., Van Seuningen I., Matria A., Montgomery E.A. (2012). Mucin 16 (cancer antigen 125) expression in human tissues and cell lines and correlation with clinical outcome in adenocarcinomas of the pancreas, esophagus, stomach, and colon. Hum. Pathol..

[122] Kim R., Emi M., Tanabe K., Toge T. (2004). Therapeutic potential of antisense BCl-2 as a chemosensitizer for cancer therapy. Cancer.

[123] Han J.P., Hong S.J., Kim H.K., Kim H.S., Lee Y.N., Lee T.H., Lee J.S. (2016). Expression of immunohistochemical markers according to histological type in patients with early gastric cancer. Scand J. Gastroenterol..

[124] Mejías-Luque R., Lindén S.K., Garrido M., Tye H., Najdovska M., Jenkins B.J., Iglesias M., Ernst M., de Bolós C. (2010). Inflammation modulates the expression of the intestinal mucins MUC2 and MUC4 in gastric tumors. Oncogene.

[125] Hwang I., Kang Y.N., Kim J.Y., Do Y.R., Song H.S., Park K.U. (2012). Prognostic significance of membrane-associated mucins 1 and 4 in gastric adenocarcinoma. Exp. Ther. Med..

[126] Baldus S.E., Mőnig S.P., Arkenau V., Hanisch F.-Z., Schneider P.M., Thiele J., Hőlscher A.H., Dienes H.P. (2002). Correlation of MUC5AC immunoreactivity with histopathological subtypes and prognosis of gastric carcinoma. Ann. Surg. Oncol..

[127] Baldus S.E., Zirbes T.K., Engel S., Hanisch F.G., Mőnig S.P., Lorenzen J., Glossmann J., Fromm S., Thiele J., Pichlmaier H. (1998). Correlation of the immunohistochemical reactivity of mucin peptide cores MUC1 and MUC2 with the histopathological subtype and prognosis of gastric carcinomas. Int. J. Cancer.

[128] Terada T. (2013). An immunohistochemical study of primary signet-ring cell carcinoma of the stomach and colorectum: II. Expression of MUC1, MUC2, MUC5AC, and MUC6 in normal mucosa and in 42 cases. Int. J. Clin. Exp. Pathol..

[129] Udhayakumar G., Jayanthi V., Devaraj N., Devaraj H. (2007). Interaction of MUC1 with beta-catenin modulates the Wnt target gene cyclinD1 in H. pylori-induced gastric cancer. Mol. Carcinog..

[130] Yonezawa S., Kitajima S., Higashi M., Osako M., Horinouchi M., Yokoyama S., Kitamoto S., Yamada N., Tamura Y., Shimizu T. (2012). A novel anti-MUC1 antibody against the MUC1 cytoplasmic tail domain: Use in sensitive identification of poorly differentiated cells in adenocarcinoma of the stomach. Gastric Cancer.

[131] Tajima Y., Yamazaki K., Nishino N., Morohara K., Yamazaki T., Kaetsu T., Suzuki S., Kawamura M., Kumagai K., Kusano M. (2004). Gastric and intestinal phenotypic marker expression in gastric carcinomas and recurrence pattern after surgery-imunohistochemical analysis of 213 lesions. Br. J. Cancer.

[132] Shinozaki E., Adachi S., Shoda J., Kawamoto T., Suzuki H., Irimura T., Ohkohchi N. (2004). Subcellular localization of MUC1 recognized by a monoclonal antibody MY.1E12 correlates with postsurgical prognosis in differentiated-type gastric carcinomas of stage II and III. Int. J. Oncol..

[133] Shimamura T., Ito H., Shibahara J., Watanabe A., Hippo Y., Taniguchi H., Chen Y., Kashima T., Ohtomo T., Tanioka F. (2005). Overexpression of MUC13 is associated with intestinal-type gastric cancer. Cancer Sci..

[134] Shi D., Qiu X.-M., Yan X.-J. (2014). The changes in MUC5AC expression in gastric cancer before and after Helicobacter pylori eradication. Clin. Res. Hepatol. Gastroenterol..

[135] Retterspitz M.F., Mőnig S.P., Schreckenberg S., Schenider P.M., Hőlscher A.H., Dienes H.P., Baldus S.E. (2010). Expression of {beta}-catenin, MUC1 and c-met in diffuse-type gastric carcinomas: Correlations with tumour progression and prognosis. Anticancer Res..

[136] Reis C.A., David L., Nielsen P.A., Clausen H., Mirgorodskaya K., Roepstorff P., Sobrinho-Simŏes M. (1997). Immunohistochemical study of MUC5AC expression in human gastric carcinomas using a novel monoclonal antibody. Int. J. Cancer.

[137] Pinto-de-Sousa J., David L., Reis C.A., Gomes R., Silva L., Pimenta A. (2002). Mucins MUC1, MUC2, MUC5AC and MUC6 expression in the evaluation of differentiation and clinico-biological behaviour of gastric carcinoma. Virchows Arch..

[138] Mariette C., Piessen G., Leteurtre E., Hémon B., Triboulet J.-P., Van Seuningen I. (2008). Activation of MUC1 mucin expression by bile acids in human esophageal adenocarcinomatous cells and tissues is mediated by the phosphatidylinositol 3-kinase. Surgery.

[139] Lin S., Zhang Y., Hu Y., Yang B., Cui J., Huang J., Wang J.M., Xing R., Lu Y. (2019). Epigenetic downregulation of MUC17 by H.pylori infection facilitates NF-κB-mediated expression of CEACAM1-3S in human gastric cancer. Gastric Cancer.

[140] Lee O.-J., Kim H.-J., Kim J.-R., Watanabe H. (2009). The prognostic significance of the mucin phenotype of gastric adenocarcinoma and its relationship with histologic classifications. Oncol. Rep..

[141] Lee H.S., Lee H.K., Kim H.S., Yang H.K., Kim Y.I., Kim W.H. (2001). MUC1, MUC2, MUC5Ac, and MUC6 expressions in gastric carcinomas: Their roles as prognostic indicators. Cancer.

[142] Kim S.M., Kwon C.H., Shin N., Park D.Y., Moon H.J., Kim G.H., Jeon T.Y. (2014). Decreased MUC5AC expression is associated with poor prognosis in gastric cancer. Int. J. Cancer.

[143] Khattab A.-Z., Nasif W.A., Lotfy M. (2011). MUC2 and MUC6 apomucins expression in human gastric neoplasm: An immunohistochemical analysis. Med. Oncol..

[144] Davison J.M., Ellis S.T., Foxwell T.J., Luketich J.D., Gibson M.K., Kuan S.-F., Nason K.S. (2014). MUC2 expression is an adverse prognostic factor in superficial gastroesophageal adenocarcinomas. Hum. Pathol..

[145] Akyűrek N., Akyol G., Dursun A., Yamaç D., Gűnel N. (2002). Expression of MUC1 and MUC2 mucins in gastric carcinomas: Their relationship with clinicopathologic parameters and prognosis. Pathol. Res. Pract..

[146] Mizoshita T., Tsukamoto T., Nakanishi H., Inada K.-I., Ogasawara N., Joh T., Itoh M., Yamamura Y., Tatematsu M. (2003). Expression of Cdx2 and the phenotype of advanced gastric cancers: Relationship with prognosis. J. Cancer Res. Clin. Oncol..

[147] Bae H.I., Li Y.-H., Na Y.K., Jung Y.W., Lee S.M., Yang J.S., Kim D.S. (2010). Overexpression of the MUC2 gene through promoter hypomethylation in mucinous cell carcinomas and signet ring cell carcinomas of gastric cancer. Genes Genom..

[148] Park K.K., Yang S.I., Seo KWYoon K.Y., Lee S.H., Jang H.K., Shin Y.M. (2015). Correlations of Human Epithelial Growth Factor Receptor 2 Overexpression with MUC2, MUC5AC, MUC6, p53, and Clinicopathological Characteristics in Gastric Cancer Patients with Curative Resection. Gastroenterol. Res. Pract..

[149] Nakashima H., Yamasaki T., Owari M., Tokai Y., Kawachi H., Sakaki N., Yoshida M. (2016). Mucin Phenotypic Expression and Submucosal Invasion of Gastric Differentiated-type Adenocarcinoma with Minimal Intestinal Metaplasia. J. Gastroenterol. Hepatol. Res..

[150] Wakatsuki K., Yamada Y., Narikiyo M., Ueno M., Takayama T., Tamaki H., Miki K., Matsumoto S., Enomoto K., Yokotani T. (2008). Clinicopathological and prognostic significance of mucin phenotype in gastric cancer. J. Surg. Oncol..

[151] Yang Y., Fang E., Luo J., Wu H., Jiang Y., Liu Y., Tong S., Wang Z., Zhou R., Tong Q. (2019). The Antioxidant Alpha-Lipoic Acid Inhibits Proliferation and Invasion of Human Gastric Cancer Cells via Suppression of STAT3-Mediated MUC4 Gene Expression. Oxid. Med. Cell Longev..

[152] Ohno T., Aihara R., Kamiyama Y., Mochiki E., Asao T., Kuwano H. (2006). Prognostic significance of combined expression of MUC1 and adhesion molecules in advanced gastric cancer. Eur. J. Cancer.

