# Supplementary material for: The Role of Mucin Expression in the Diagnosis of Oesophago-Gastric Cancer: A Systematic Literature Review
Source: Cancers (Basel). 2023 Nov 1;15(21):5252. doi: 10.3390/cancers15215252 (PMC10650431; doi:10.3390/cancers15215252)
Supplement: Supplementary file 1 [file cancers-15-05252-s001.zip › cancers-2632161-supplementary.pdf]

| Author | Year | Total score | Author | Year | Total score | Author | Year | Total score |
|---|---|---|---|---|---|---|---|---|
| Aihara et al. | 2005 | 3 | Han et al | 2009 | 6 | Song et al | 2021 | 8 |
| Ando et al. | 2009 | 3 | He et al | 2017 | 5 | Sugai et al | 2004 | 8 |
| Babu et al. | 2006 | 3 | Javanbakht et al | 2017 | 6 | Sun et al | 2016 | 8 |
| Benjamin et al | 2010 | 4 | Jia et al | 2010 | 6 | Tajima et al | 2007 | 5 |
| Boltin et al | 2014 | 6 | Kageyama-Yahara et al | 2014 | 6 | Tamura et al | 2012 | 8 |
| Burjonrappa et al | 2007 | 4 | Khor et al | 2020 | 6 | Tsukashita et al | 2001 | 6 |
| Butt et al | 2017 | 1 | Lee et al | 2007 | 6 | Wang et al | 2003 | 8 |
| Chaves et al | 2005 | 6 | Leteurtre et al | 2006 | 8 | Wang et al | 2015 | 8 |
| Cheah et al | 1994 | 1 | Li et al | 2015 | 8 | Xiong et al | 2017 | 8 |
| Chlumska et al | 2018 | 3 | Li et al | 2008 | 8 | Yamada et al | 2020 | 8 |
| Cho et al | 1991 | 3 | Machado et al | 2000 | 5 | Endo et al | 1998 | 5 |
| Choi et al | 2009 | 5 | Mall et al | 2000 | 5 | Higuchi et al | 2005 | 7 |
| Conze et al | 2010 | 4 | Myllykangas et al | 2008 | 5 | Yu et al | 2016 | 8 |
| Cui et al | 2015 | 4 | Nakajima et al | 2003 | 6 | Tian et al | 2012 | 8 |
| Cui et al | 2021 | 5 | Ozcan et al | 2018 | 5 | Setia et al | 2016 | 7 |
| DiMaio et al | 2012 | 5 | Palmer et al | 2012 | 5 | Gurbuz et al | 2004 | 8 |
| Flucke et al | 2003 | 6 | Piessen et al | 2007 | 4 | Wang et al | 2000 | 8 |
| Forné et al | 2009 | 6 | Pinto de Sousa et al | 2004 | 8 | Semino-Mora et al | 2003 | 8 |
| Fujita et al | 2020 | 4 | Puyan et al | 2011 | 6 | Fan et al | 2009 | 8 |
| Guillem et al | 2000 | 6 | Reis et al | 1998 | 8 | Buisine et al | 1998 | 1 |
| Guner et al | 2018 | 6 | Sakamoto et al | 1997 | 8 | Weimann et al | 2010 | 1 |
| Gurbuz et al | 2002 | 5 | Shi et al | 2021 | 8 | Takami et al | 2012 | 6 |

| Author | Year | Total score | Author | Year | Total score | Author | Year | Total score |
|---|---|---|---|---|---|---|---|---|
| Begnami et al | 2011 | 1 | Baldus et al | 2002 | 7 | Lee et al | 2008 | 6 |
| Ushiku et al | 2013 | 7 | Baldus et al | 1998 | 7 | Lee et al | 2001 | 8 |
| Choi et al | 2009 | 7 | Terada et al. | 2013 | 7 | Kim et al | 2013 | 6 |
| Szachnowicz et al | 2008 | 7 | Udhayakumar et al | 2007 | 7 | Khattab et al | 2010 | 4 |
| Zhang et al | 2004 | 7 | Gurbuz et al | 2004 | 7 | Davison et al | 2014 | 6 |
| Kim et al | 2013 | 7 | Wang et al | 2000 | 8 | Akyürek et al | 2001 | 6 |
| Wang et al | 2003 | 6 | Semino-Mora et al | 2003 | 8 | Mizoshita et al | 2003 | 6 |
| Mitsuuchi et al | 1999 | 7 | Yonezawa et al | 2011 | 4 | Bae et al | 2010 | 4 |
| Kang et al | 2012 | 8 | Tajima et al | 2004 | 7 | Park et al | 2015 | 8 |
| Dwertmann et al | 2021 | 8 | Sun et al | 2018 | 7 | Nakashima et al | 2016 | 6 |
| Gulmann et al | 2003 | 8 | Song et al | 2002 | 6 | Chinyama et al | 1999 | 7 |
| Fujimoto et al | 2017 | 8 | Shinozaki et al | 2004 | 6 | Wakatsuki et al | 2008 | 7 |
| Ge et al | 2020 | 7 | Shimamura et al | 2005 | 5 | Yang et al | 2019 | 7 |
| Ide et al | 2012 | 7 | Shi et al | 2013 | 6 | Piessen et al | 2009 | 6 |
| Streppel et al | 2012 | 7 | Retterspitz al | 2010 | 6 | Ohno et al | 2006 | 6 |
| Kim et al | 2004 | 8 | Reis et al | 1997 | 6 | | | |
| Han et al | 2015 | 7 | Reis at al | 2000 | 4 | | | |
| Mejı´ as-Luque et al | 2010 | 7 | Pinto-de-Sousa et al | 2002 | 4 | | | |
| Mesquita et al | 2002 | 7 | Mariette et al | 2008 | 4 | | | |
| Hwang et al | 2012 | 7 | Lin et al | 2019 | 8 | | | |

**Table S1: Total Newcastle-Ottawa quality assessment scores for all included studies.**
